# Role of the Gas6/TAM System as a Disease Marker and Potential Drug Target

**DOI:** 10.1155/2021/2854925

**Published:** 2021-01-13

**Authors:** Pier Paolo Sainaghi, Mattia Bellan, Alessandra Nerviani

**Affiliations:** ^1^Department of Translational Medicine, Università del Piemonte Orientale UPO, Novara, Italy; ^2^Division of Rheumatology, CAAD (Center for Translational Research on Autoimmune and Allergic Diseases) “AOU Maggiore della Carità”, Novara, Italy; ^3^IRCAD, Interdisciplinary Research Center of Autoimmune Diseases, Novara, Italy; ^4^Experimental Medicine and Rheumatology, William Harvey Research Institute and Barts and The London School of Medicine and Dentistry, Queen Mary University of London, London, UK

Growth arrest-specific 6 (Gas6) is a gene cloned in 1993 [1] encoding for a vitamin K-dependent protein expressed in different tissues [1–3]. Its biological activities are mediated by the interaction with three tyrosine kinase receptors: Tyro3, Axl, and MerTK, which are commonly and collectively abbreviated as TAM [4]. These receptors share a common feature: their extracellular domain is proteolytically cleaved and released in a soluble form (sTyro3, sAxl, and sMer; sTAM collectively) and acts as a decoy receptor; consistently, the shedding of the ectodomain entails the reduction of transmembrane receptors available for the ligands [5, 6].

Different activities have been attributed to Gas6/TAM interaction: it has been shown to act on platelet function [7], to regulate cell growth [8], to mediate the phagocytosis of apoptotic bodies [9], and to switch off inflammatory response [10].

On these bases, Gas6 and sTAM role as biomarkers has been explored and partially validated in several human diseases, particularly in those where fibrosis and inflammation are relevant [11]. In this context, the diagnostic performance of Gas6 has been evaluated in neuroinflammatory [12–14] and neurodegenerative disorders [15]; moreover, Gas6 and the circulating forms of TAM receptors have been observed to be increased in the plasma of patients affected by systemic lupus erythematosus, being predictive of disease severity [16, 17]. Among other inflammatory disorders, Gas6 and TAM system receptors have been also proposed as disease biomarkers in rheumatoid arthritis [18] and Sjogren's syndrome [19].

Consistently, being related to fibrosis and inflammation, Gas6 and sAxl have been found increased in the plasma of patients affected by liver cirrhosis [20], a condition in which the overly exuberant accumulation of extracellular matrix proteins commonly triggered by chronic injury of the hepatic parenchyma with an inflammatory component leads to hepatic fibrosis with a structural and functional disruption. In this context, since their plasmatic levels are predictive of the development of complications of chronic liver diseases such as hepatocellular carcinoma [21] and oesophageal varices [22], they may be proposed as biomarkers of disease severity [23].

We should not neglect that Gas6 has a great structural homology with protein S, an important regulator of coagulative cascade, which also shares the same receptors. Therefore, the system has been explored in the context of thromboembolic but its role in clinical setting is under investigation [24, 25].

Finally, an overactivation of the system has been associated to several solid and hematological neoplastic conditions and identified as a potential negative prognostic biomarker [26–29].

However, in all these conditions, the altered plasmatic concentration of Gas6 and its receptors does not seem to be only an epiphenomenon, but rather to contribute to disease pathogenesis. This is why, targeting TAM is a novel strategy proposed for different human diseases. Recently, Espindola et al. [30] have demonstrated that both Gas6 and Axl expressions are enhanced in patients with idiopathic pulmonary fibrosis (IPF); interestingly, specifically targeting Gas6/Axl interaction significantly inhibited the synthetic, migratory, and proliferative properties of IPF fibroblasts and prevented the development of pulmonary fibrosis in a murine model. Consistently, the blockade of Gas6/Axl axis is associated to a reduced collagen deposition and liver fibrosis in a murine model of Ccl-4-induced liver disease [31]. These findings support the idea that Gas6/TAM system is a promising target of antifibrotic treatments. This is not surprising, considering that tyrosine kinase inhibition is a common strategy in oncology as well; consistently, TAM receptor blockade has been already proposed for different neoplastic conditions [32–35].

In conclusion, a deeper knowledge of this relatively novel and unexplored system might contribute to clarify the pathogenetic mechanisms underlying the development of different human diseases and, potentially, to make available novel promising therapeutic tools.

*Pier Paolo Sainaghi* *Mattia Bellan* *Alessandra Nerviani*
